# Affective and cognitive impairments in patients with epilepsy

**DOI:** 10.1192/j.eurpsy.2021.1099

**Published:** 2021-08-13

**Authors:** I. Blazhina, V. Korostiy

**Affiliations:** 1 Department Of Nervous Diseases Psychiatry And Medical Psychology, Bucovinian State Medical University, Chernivtsi, Ukraine; 2 Psychiatry, Narcology, Medical Psychology And Social Work, Kharkiv National Medical University, Kharkiv, Ukraine

**Keywords:** Epilepsy, Cognitive disorders, Affective disorders

## Abstract

**Introduction:**

The most common psychiatric conditions in epilepsy are depression, anxiety, behavioral, psychotic disorders and cognitive disorders as well as those which can be caused by convulsive seizures.

**Objectives:**

The aims of the research were to define cognitive and affective impairments in patients with epilepsy and their quality of life. Since the presents of cognitive impairments and affective disorders have a considerable impact on the functioning of patients, their socialization and the level of their disability.

**Methods:**

We studied the features of clinical and psychopathological manifestations in patients suffering from epilepsy. The study covered 100 patients (47 men and 53 women) who were in inpatient care. The following psychodiagnostic techniques were used: the test of 10 words of Luria, the MOCA test, the Münsterberg test, Mini-Mult test, the quality of life scale, the Hamilton scale of depression and anxiety.

**Results:**

The following data of the study were observed: 88 % patients had decreased memory, 38% had symptoms of depression, 28% had mild situational or neurotic depression, 8% had moderate depression, 2% had severe depression, 20% had a state of severe anxiety, 16% had symptoms of anxiety. The average rate of quality of life among all examined people was 67.5 out of 100.

**Conclusions:**

The results of the conducted research indicate the need for further study of the features of the comorbid pathology in epilepsy and development and implementation pharmacological and nonpharmacological methods for treatments of epilepsy.

